# Impact of genetically engineered maize on agronomic, environmental and toxicological traits: a meta-analysis of 21 years of field data

**DOI:** 10.1038/s41598-018-21284-2

**Published:** 2018-02-15

**Authors:** Elisa Pellegrino, Stefano Bedini, Marco Nuti, Laura Ercoli

**Affiliations:** 1 Institute of Life Sciences, Scuola Superiore Sant’Anna, Piazza Martiri della Libertà 33, 56127 Pisa, Italy; 2 Department of Agriculture, Food and Environment, University of Pisa, via del Borghetto 80, 56125 Pisa, Italy

## Abstract

Despite the extensive cultivation of genetically engineered (GE) maize and considerable number of scientific reports on its agro-environmental impact, the risks and benefits of GE maize are still being debated and concerns about safety remain. This meta-analysis aimed at increasing knowledge on agronomic, environmental and toxicological traits of GE maize by analyzing the peer-reviewed literature (from 1996 to 2016) on yield, grain quality, non-target organisms (NTOs), target organisms (TOs) and soil biomass decomposition. Results provided strong evidence that GE maize performed better than its near isogenic line: grain yield was 5.6 to 24.5% higher with lower concentrations of mycotoxins (−28.8%), fumonisin (−30.6%) and thricotecens (−36.5%). The NTOs analyzed were not affected by GE maize, except for Braconidae, represented by a parasitoid of European corn borer, the target of Lepidoptera active Bt maize. Biogeochemical cycle parameters such as lignin content in stalks and leaves did not vary, whereas biomass decomposition was higher in GE maize. The results support the cultivation of GE maize, mainly due to enhanced grain quality and reduction of human exposure to mycotoxins. Furthermore, the reduction of the parasitoid of the target and the lack of consistent effects on other NTOs are confirmed.

## Introduction

Since their first commercialization in 1996^1,2^, genetically engineered (GE) crops have been rapidly adopted in many countries^3^ becoming the fastest adopted crop technology in the world^4^. GE crop cultivation has increased from 1.7 million hectares in 1996 to 185.1 million hectares in 2016, representing about 12% of the global cropland, 54% of which are found in developing countries^4^.

In 2016, the different GE traits introduced into major crops (soybean, maize, canola, and cotton) consist of herbicide tolerance (HT) which comprise 95.9 million hectares of GE crops (53% of the total GE area); insect resistance (IR) at 25.2 million hectares (14% of the total GE area) and both HT and IR stacked in one crop, at 58.5 million hectares (33% of the total GE area)^4^.

Despite the extensive cultivation of GE crops and a considerable number of scientific reports, the concerns about their safety has led 38 countries worldwide, including 19 in Europe, to officially prohibit their cultivation, though allowing the import of food and feed derived from or consisting of GE plants^4^.

Among GE crops, maize (*Zea mays* L.) has the highest number of approved events (single and stacked traits) and is the second largest crop, after soybean, in terms of global adoption^5^. In 2015, 53.6 million ha of GE maize were cultivated on a global scale, representing almost 1/3 of the 185 million ha of maize planted worldwide. Thirty-three million ha were grown in USA, while GE maize planted in Brazil, Argentina, and Canada accounted for 17.4 million ha. The global value of GE maize is estimated at US$ 8.1 billion^4^. Moreover, among GE crops, maize has the highest potential of expansion. This is due to its comparatively low rate of adoption (30% of the global maize in 2015) and its huge cultivated area^4^.

Numerous attempts have been carried out to synthesize the huge literature on agronomic and economic performance and environmental impact of GE maize (*e.g*.,^6–13^). However, these studies, mostly literature reviews, do not allow us to draw univocal conclusions.

To date, a few meta-analyses have been performed on GE maize at farm and field level addressing questions concerning yield, production cost and gross margin terms^14–16^, pesticide use^16^, and effects on non-target (NT) invertebrates^17–20^. However, there are still some unsettled key issues in GE maize cultivation which remain to be addressed, such as if GE technology improves the grain quality in terms of nutritional value and toxin content (including mycotoxins)^21,22^, and if it affects important agro-ecosystem services including soil organic matter decomposition.

Therefore, this study is aimed at increasing our knowledge about agronomic traits and safety for human health and environment of GE maize cultivation by performing a meta-analysis of the peer-reviewed literature (from 1996 to 2016) on yield and by extending the analysis on new parameters, such as grain quality, non target organisms (NTOs) at family level, target organisms (TOs) and soil biomass decomposition, allowing more robust evaluation of the field performance of GE maize. This study, embracing the period 1996–2016, applies rigorous criteria for study selection, such as the inclusion in the dataset of field observations comparing GE maize with its true non-GE isoline or near isoline, throughout its overall cultivation period.

## Results

### Composition of the database

**Figure 1:**
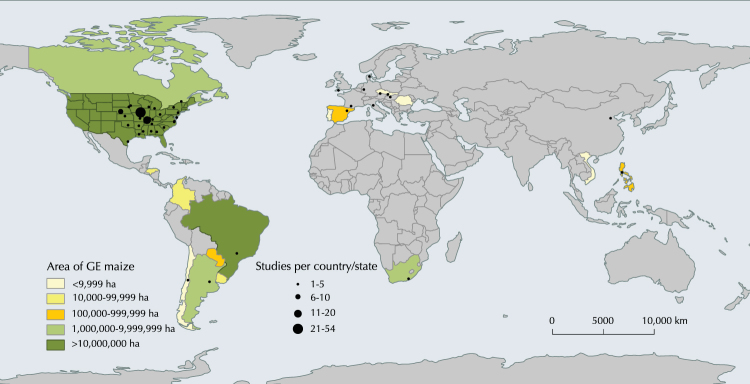
Worldwide distribution of the field studies reported in the articles selected for the meta-analysis. Area of GE maize cultivation by country in 2016 is indicated in the map (data from^4^). Map generated using the open source QGIS ver. 2.18.9. (QGIS Development Team, 2016. QGIS Geographic Information System. Open Source Geospatial Foundation. http://qgis.osgeo.org).

The first step of the selection procedure yielded 6,006 publications. The subsequent refinement, by adopting the stringent criteria above described, gave 32, 5, 32 and 10 eligible publications, covering, respectively, the following categories: grain yield and quality, TOs, NTOs (non-target organisms), and biogeochemical cycles (*e*.*g*. lignin content in stalks and leaves, stalk mass loss and biomass loss, CO_2_ emission) (Supplementary 1 Tables 1–5). The comparison of our dataset with the available NTO dataset of Wolfenbarger *et al*.^18^ allowed the inclusion of 40 observations (Supplementary 4). The main reasons for paper exclusion were that the experiments were not performed under field conditions, they did not have a near isogenic hybrid as comparator, hybrids were not grown under identical conditions, or the data lacked of a measure of variance, statistical significance, or sample size. For the traits within each category, the maximum number of observations has been taken into account. No papers were selected for the biodiversity category and for CO_2_ emission trait. The number of papers and observations utilised for the meta-analysis of each trait is reported in Table 1. Overall, grain yield and quality, TOs, NTOs and biogeochemical cycle databases were composed of 542, 99, 813, and 29 observations, respectively (see databases provided as Supplementary 2, 3, 4, and 5). Regarding the geographic distribution of the field studies, the majority of them were performed in North America (202), followed by Europe (52), and South America (17) (Supplementary 1 Tables 2–5). Asia, Africa and Australia were represented with eleven, one, and no studies, respectively (Fig. 1). Ninety-nine per cent of the studies in North America were done in the United States, of which 49% in Iowa, Illinois, and Nebraska. In Europe the field studies were performed in nine countries (Germany, Spain, France, the Czech Republic, Denmark, Hungary, Italy, Slovakia, and the United Kingdom), while in South America they were performed in three countries (Brazil, Argentina, and Chile).

**Table 1:** Number of studies and observations for the analyzed traits.

Impact type	Trait	Number of studies	Number of observations
Grain yield	Grain yield-all	19	276
	Grain yield-single stack	16	126
	Grain yield-double stack	10	92
	Grain yield-triple stack	3	36
	Grain yield-quadruple stack	2	22
Damaged ears	Damaged ears-all	7	139
	Damaged ears-single stack	4	37
	Damaged ears-double stack	3	42
	Damaged ears-triple stack	2	36
	Damaged ears-quadruple stack	2	24
Grain quality	Proteins	3	6
	Lipids	3	6
	Acid Detergent Fiber	3	6
	Neutral Detergent Fiber	3	6
	Total Detergent Fiber	3	6
	Fumonisins	4	20
	Thricotecens	4	22
	Mycotoxins	9	55
TO taxa	Diabrotica spp. (adults)	5	99
NTO taxa	Anthocoridae (adults)	15	80
	Aphididae (adults)	8	59
	Araneae (adults)	12	104
	Braconidae (adults)	4	105
	Carabidae (adults)	10	88
	Chrysopidae (adults)	9	53
	Chrysopidae (larvae)	3	7
	Cicadellidae (adults)	6	23
	Coccinellidae (adults)	17	164
	Coccinellidae (larvae)	4	9
	Nabidae (adults)	5	17
	Nitidulidae (adults)	6	50
	Staphylinidae (adults)	9	54
Biomass decompostition	Lignin in leaves	3	4
	Lignin in stalks	3	4
	Stalk mass loss	3	7
	Residue mass loss	3	6
	CO_2_ emission	3	8

Grain yield response was based on 46% of observations on single event hybrid maize, and on 33, 13, and 8% of double, triple and quadruple stacks (GE events combined by hybridisation) in hybrid maize, respectively (Table 2).

**Table 2:** Number of observations for the analyzed traits in stacked maize hybrids.

	Single event	Double stack	Triple stack	Quadruple stack
Grain Yield				
Lepidopteran resistance	125			
Phytase enzyme (phyA2)	1			
Lepidopteran resistance and glufosinate tolerance		84		
Lepidopteran resistance		8		
Lepidopteran and coleopteran resistance, and glyphosate tolerance			26	
Lepidopteran resistance and glufosinate tolerance			8	
Lepidopteran and insect resistance, and glufosinate tolerance			2	
Coleopteran and lepidopteran resistance, and glyphosate tolerance				18
Coleopteran and lepidopteran resistance, and glufosinate tolerance				4
Total	126	92	36	22
Damaged ears				
Lepidopteran resistance	37	8		
Lepidopteran resistance and glufosinate tolerance		34		
Lepidopteran and coleopteran resistance, and glyphosate tolerance			28	
Lepidopteran and coleopteran resistance, and glufosinate tolerance			8	
Coleopteran and lepidopteran resistance, and glyphosate tolerance				20
Coleopteran and lepidopteran resistance, and glufosinate tolerance				4
Total	37	42	36	24
TO				
Coleopteran resistance	44			
Insect resistance	20			
Insect and coleopteran resistance		20		
Insect and coleopteran resistance, and herbicide tolerance		15		
Total	64	35		

The damaged ear dataset was composed of damage data caused by the fall armyworm, *Spodoptera frugiperda* (J.E. Smith) (Lepidoptera: Noctuidae) and the corn earworm, *Helicoverpa zea* (Boddie) (Lepidoptera: Noctuidae), and by the bacterium *Dickeja dadantii* (Samson) (formerly named as *Erwinia chrysanthemi* pv. *zeae*) (Enterobacteriales: Enterobacteriaceae) (Supplementary 2). The response of damaged ears was based on observations on single (27%), double (30%), triple (26%) and quadruple (17%) stacked hybrids (Table 2).

The TO dataset was composed of data on the abundance of *Diabrotica* spp.; western corn rootworm, *Diabrotica virgifera virgifera* (LeConte); northern corn rootworm, *Diabrotica barberi* (Smith and Lawrence), and southern corn rootworm, *Diabrotica undecimpuctata howardi* (Mannerheim) (Coleoptera: Chrysomelidae). For species other than *Diabrotica* spp. there were not enough observations to perform meta-analysis. The TO dataset was analyzed at genus level to achieve an adequate sample size for analyses (Supplementary 3). The response of *Diabrotica* spp. was based on 65% of observations on single hybrids, and 35% on double hybrids (Table 2).

The NTO datasets were composed of data on the abundances of NTOs classified as phylum, class, order, and family levels, but they were analyzed at family level (Supplementary 1 Table 4; Supplementary 4) that was the finest possible taxonomic resolution allowing reliable analyses. The analyzed NTO families were: Anthocoridae, Aphididae, Braconidae, Carabidae, Chrysopidae, Cicadellidae, Coccinellidae, Nabidae, Nitidulidae, and Staphylinidae (Supplementary 1 Table 4). Spiders were analyzed at the order level Araneae. For the response of all families the abundance of adults was analyzed, whereas for Chrysopidae and Coccinellidae the abundance of larvae was also analyzed,

Details about number of observations for the traits grain yield, damaged ears and TOs in single, double, triple and quadruple stacked hybrids are given in Table 2.

### Effects on grain yield and quality

**Figure 2:**
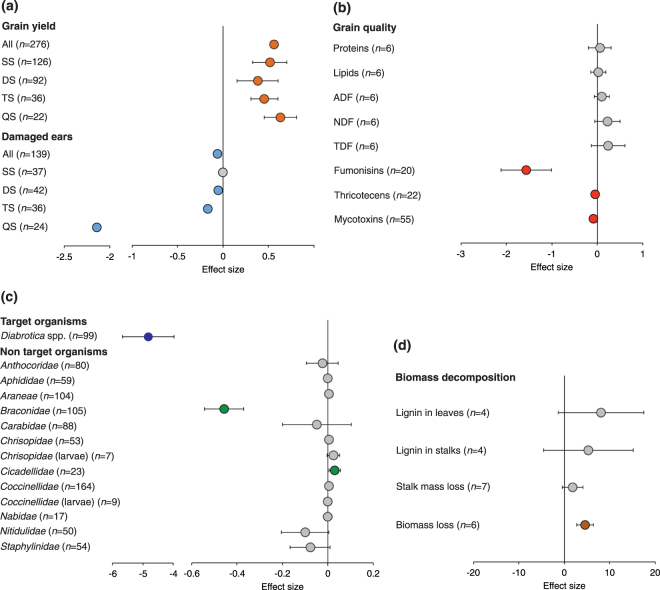
Effect sizes. Effects of GE maize hybrids on grain yield and damaged ears (**a**), grain quality (**b**), target organism (*Diabrotica* spp.) and non-target organisms (**c**) and biomass decomposition (**d**). Effect size was calculated by weighted Hedge’s *g* (g^+^). Bars around the means indicate 95% bootstrap confidence intervals (CIs). A mean effect size is significantly different from zero when its 95% CI does not overlap zero. Positive and negative g^+^ imply an increase and decrease in the trait compared with the maize isoline, respectively. Numbers in parentheses indicate the number of observations for each trait. In (**c**) when not specified, target and non-target organism(s) are considered as adult insects. SS = single event hybrid; DS = double stacked hybrid; TS = triple stacked hybrid; QS = quadruple stacked hybrid; ADF = Acid Detergent Fiber; NDF = Neutral Detergent Fiber; TDF = Total Detergent Fiber.

**Figure 3:**
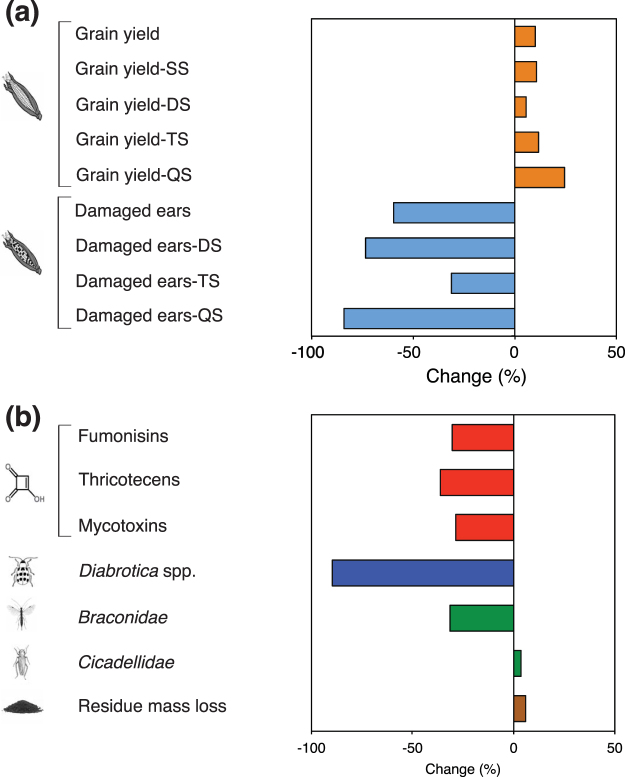
Response ratios. Effects of GE maize hybrids on the significant traits: grain yield and damaged ears (**a**), grain quality (fumonisins, thricotecenes, mycotoxins), target (*Diabrotica* spp.) and non-target (*Braconidae*, *Cicadellidae*) organisms and residue mass loss (**b**). The response ratio was calculated as the mean percentage of change for the weighted Hedge’s *g* (g^+^) values different from zero between the GE hybrids and their isolines. SS = single eventhybrid; DS = double stacked hybrid; TS = triple stacked hybrid; QS = quadruple stacked hybrid.

Almost all studies on grain yield and damaged ears were done with plants expressing *cry* genes against Lepidoptera, some also stacked with genes against Coleoptera and herbicide-tolerance (Table 2). Genetically engineered maize cultivation led to a significant increase of yield compared to the non-GE isolines or near isolines. The overall mean effect size (g^+^) for grain yield was 0.526 (Fig. 2; Supplementary 1 Table 6) and the percentage of change was 10.1% (Fig. 2). The mean effect sizes calculated for the grain yield of single, double, triple and quadruple stacked hybrids were positive (g^+^ ranging from 0.38 to 0.629) and significant (Fig. 2; Supplementary 1 Table 6). The percentage of change ranged from 5.6 to 11.7% for single, double and triple stacked hybrids, while it was 24.5% for quadruple staked hybrids (Fig. 3). Mean effect size responses did not significantly change when we accounted for another sensitivity test based on the removal of a set of observations (reduced dataset) and the results were also robust to publication biases (Table 3; Supplementary 1 Table 6). The publication biases were assessed by the sensitivity analysis that compares the fail-safe number (N) with a threshold calculated as 5n + 10, where n is the original number of studies. A fail-safe number is considered robust if it is greater than the threshold value.

**Table 3:** Sensitivity analysis based on the fail-safe number (i.e., the additional number of observations necessary to change results of the meta-analysis from significant to non-significant) and the number of studies (n).

Impact type	Parameter	Fail-safe number	n	5n + 10
*Grain yield and quality*	Grain yield	1660287	19	105
	Grain yield-single stack	20671	16	90
	Grain yield-double stack	12002	10	60
	Grain yield-triple stack	359	3	25
	Grain yield-quadruple stack	528	2	20
	Damaged ears	103216	7	45
	Damaged ears-single stack	2360	4	30
	Damaged ears-double stack	30384	3	25
	Damaged ears-triple stack	801	2	20
	Damaged ears-quadruple stack	4763	2	20
	Proteins	0	3	25
	Lipids	9642	3	25
	Acid Detergent Fiber	0	3	25
	Neutral Detergent Fiber	0	3	25
	Total Detergent Fiber	0	3	25
	Fumonisins in grain	1596	4	30
	Thricotecens in grain	110	4	30
	Mycotoxins	7287	11	65
*TO taxa*	Diabrotica spp. (adults)	26937	5	35
*NTO taxa*	Anthocoridae (adults)	0	15	85
	Aphididae (adults)	0	8	50
	Araneae (adults)	32	12	70
	Braconidae (adults)	6114	4	30
	Carabidae (adults)	0	10	60
	Chrysopidae (adults)	0	9	55
	Chrysopidae (larvae)	22	3	25
	Cicadellidae (adults)	0	6	40
	Coccinellidae (adults)	220	17	95
	Coccinellidae (larvae)	0	4	30
	Nabidae (adults)	0	5	35
	Nitidulidae (adults)	0	6	40
	Staphylinidae (adults)	93	9	55
*Biomass decomposition*	Lignin in leaves	3227	3	25
	Lignin in stems	6216	3	25
	Stalk mass loss	5	3	25
	Biomass loss	40	3	25
	CO_2_ emission	81	3	25

The mean effect size (g^+^) of the damaged ears calculated for all hybrids was −0.061, and the 95% CI did not overlap zero (Fig. 2; Supplementary 1 Table 6). The percentage of reduction was 59.6% (Fig. 3). When differentiated into hybrid type, the mean effect size was positive for the damaged ears of double, triple and quadruple stacked hybrids, whereas the response was not significant when restricted to single hybrids (95% CI, −0.012–0.011) (Fig. 2; Supplementary 1 Table 6). The percentage of change of damaged ears was 73.4, 31.1, and 84.0%, for double, triple and quadruple stacked hybrids, respectively. These responses did not change following the sensitivity analysis based on one set of observations removal and were also supported by the fail-safe number test (Table 3; Supplementary 1 Table 6).

The concentration of proteins, lipids, Acid Detergent Fiber (ADF), Neutral Detergent Fiber (NDF) and Total Dietary Fiber (TDF) in grain did not vary between GE hybrids and the isolines or near isolines (Supplementary 1 Table 6). These responses did not change after the sensitivity analysis based on one set of observations removal (Table 3; Supplementary 1 Table 6). Only the results on lipids were supported by fail-safe number test.

Observations on mycotoxins were mostly done on plants expressing resistance to Lepidoptera (84%), while the observations on fumonisin and thricotecens were done only on plants expressing resistance to Lepidoptera. Grain concentration of mycotoxins, calculated for all hybrids, and of fumonisins and thricotecens, calculated for single stacked Cry1Ab hybrids, were significantly reduced in GE maize, with mean effect sizes ranging from −1.559 to −0.001 for fumonisins and thricotecens, respectively (Fig. 2; Supplementary 1 Table 6). The reductions ranged from 28.8 to 36.5% for mycotoxins and thricotecens, respectively (Fig. 3). These responses did not change according to the sensitivity analysis based on the removal of one set of observations and to the fail-safe number test (Table 3; Supplementary 1 Table 6).

### Effects on target and non-target organisms

Almost all observations on the abundance of TOs were done with plants expressing resistance to Coleoptera (Table 2). The abundance of *Diabrotica* spp. was highly sensitive to GE cultivation with −5.007 mean effect size and 89.7% reduction (Figs 2 and 3; Supplementary 1 Table 6). This result was supported by both sensitivity analyses (Table 3; Supplementary 1 Table 6).

The observations on the abundance of NTOs were obtained from plants expressing resistance to Coleoptera (35%) and Lepidoptera (65%) (Table 4). Among taxonomic groups of NTOs, Anthocoridae, Aphididae, Araneae, Carabidae, Chrysopidae (adults and larvae), Coccinellidae (adults and larvae), Nabidae, Nitidulidae and Staphylinidae were not affected by GE cultivation. These results were supported by the sensitivity analysis based on the removal of one set of observations (Table 3; Supplementary 1 Table 6). Only the results on Coccinellidae (adults) and Staphylinidae were supported by the fail-safe number test. By contrast, Braconidae were significantly decreased (g ^+^ = −0.457), and Cicadellidae were significantly increased (g ^+^ = 0.030) (Fig. 2; Supplementary 1 Table 6). The results on Braconidae were robust to both sensitivity tests (Supplementary 1 Tables 7 and 8). The results on Cicadellidae were not supported by sensitivity analysis (Table 3; Supplementary 1 Table 6). The abundance of Braconidae was reduced in GE maize by 31.5%, (Fig. 3).

**Table 4:** Number of observations for the analyzed traits in maize hybrids.

NTO taxa	Lepidopteran resistance	Coleopteran resistance	Lepidopteran resistance plus herbicide tolerance	Coleopteran resistance plus herbicide tolerance
Anthocoridae	17	17	26	20
Aphidae	15	12	28	4
Araneae	21	36	23	24
Braconidae	3	12	90	0
Carabidae	33	46	9	0
Chrysopidae	11	12	21	9
Chrysopidae larvae	4	0	3	0
Cicadellidae	8	0	4	11
Coccinellidae	111	0	33	20
Coccinellidae larvae	5	0	4	0
Nabidae	11	0	6	0
Nitidulidae	10	24	2	14
Staphylinidae	13	26	15	0

### Biomass decomposition

All observations were done with hybrids expressing insect resistance, either single or stacked with herbicide tolerance (Supplementary 5). Lignin concentration in leaves and stems did not change between GE hybrids and their isolines or near isolines (Fig. 2; Supplementary 1 Table 6). These results were supported by the fail-safe number test, but not by the sensitivity analysis based on the removal of one set of observations, probably due to the small number of pairwise comparisons (*n = *4) (Table 3; Supplementary 1 Table 6). Similarly, the stalk mass loss was not significantly different in GE hybrids compared to their isolines or near isolines, whereas the biomass loss, including all crop residues, was significantly increased in GE hybrids (Fig. 2; Supplementary 1 Table 6). The biomass loss corresponded to an increase of the biomass decomposition rate of 5.9% (Fig. 3). The results on biomass loss were supported by both sensitivity analyses (Table 3; Supplementary 1 Table 6).

## Discussion

### Composition of the database

To date, a considerable number of scientific articles on GE maize is present in the literature (6,006 publications examined). However, on the basis of the criteria adopted for data selection, only 76 publications were eligible for the meta-analyses. This selection suggests there is a need for more field research with a wider geographic coverage and having appropriate comparators and field design allowing robust statistical analyses. It is interesting to note that in Europe there is a relatively large number of field studies carried out in several European Union member states despite GM maize is extensively cultivated only in Spain, due to the national legislative constraints in the other countries. Moreover, there is a need to publish research data in a more standardized way, *e.g*. providing raw data with at least three replicates, allowing the calculation of variance. As regards the partitioning of GE hybrids by trait in the grain yield dataset, we noted that single event HT hybrids were missing and this did not allow the evaluation of such a major category of maize GE hybrids on grain yield and the other agro-environmental traits linked to the development of weed resistance to herbicides. Finally, we noted that some categories were not adequately covered in our database, such as biodiversity and soil biogeochemical cycles that are the processes that modulate the provision of agro-ecosystem services^23^.

### Effects on grain yield

Our study indicated that GE maize hybrids increased yield by 10.1%, corresponding to 0.7 t ha^−1^, calculated on the average grain yield of the GE isolines or near isolines in the dataset. These results, based on a high number of observations (n = 276), essentially confirmed previous results^15^, showing a GE maize yield increase of 0.6 t ha^−1^. In a meta-analysis of the yield responses of GE maize hybrids in Spain^14^, similar yield increases were recorded (5.6% corresponding to 0.7 t ha^−1^), and higher yields were reported in Germany and South Africa (12.2 and 24.6%, corresponding to 1.1 and 1.8 t ha^−1^, respectively). The yield increase for GE maize, calculated by disaggregating data reported by Klümper and Qaim^16^, was 18.1%. This higher yield compared to our results (18.1% *vs* 10.1%) might be caused by the fact that Klümper and Qaim included book chapters, grey literature and other datasets that were excluded for our meta-analysis. Indeed, this is supported by the observation that the type of publication (*i*.*e*. studies published in peer-reviewed or non-peer reviewed journals) affected the outcome of the analysis^16^. In our study we found that yield increase of GE maize varied in relation to the type of hybrid, ranging from 5.6 to 24.5% in double and quadruple stacked hybrids, respectively. Quadruple stacked hybrids provided higher grain yields. This could be related to a greater overall pest protection due to the insertion of multi-events providing resistance to Coleoptera and Lepidoptera^10,24^, confirming the positive outcome of the new genetic-engineering technologies^13^. Global losses of maize production due to pests and weeds are estimated at 31.2% and 10.5%, respectively^25^, while the yield gain provided by insect pest management by chemical insecticides is estimated about 18%^26^.

### Effects on crop protection chemicals

Due to the selection criteria adopted, in our study we did not find a sufficient number of data for analyzing the quantity of insecticide and herbicide utilized in GE maize compared to the isolines or near isolines and for performing an economic analysis. Other authors have estimated that in the period from 1996 to 2011 the adoption of GE HT and IR maize caused a reduction in the volume of the active ingredient of herbicides and insecticides of 10.1% and 45.2%, respectively^27^. According to this study, the adoption of GE HT crops resulted mainly in a shift of the profile of used herbicides, and the GE IR technology has effectively reduced insecticides used to control important crop pests.

Previous meta-analyses compared Bt crops and non-Bt crops that had been treated with insecticides^17,18,20^. These studies indicated that the systems using GE technology have benefited also from better biological control of all the pests the technology does not affect. This could be considered an indirect benefit of the technology.

### Effects on quality traits

The results clearly indicate that GE maize grain contains lower amounts of mycotoxins (29%), fumonisin (31%) and thricotecens (37%) than its non-GE counterpart. The lower mycotoxin content seems to be related to the lower incidence of insect attack, since GE maize resulted in 59.6% less damaged ears compared to the corresponding isolines or near isolines. Insects promote fungal colonization by acting as vectors of fungal spores and by creating wounds in kernels on which the germination of fungal spores is favoured during cultivation and storage with resultant mycotoxin accumulation in grain^28–30^. Mycotoxins are toxic and carcinogenic for humans and animals, and the high mycotoxin content in grain, beside the health risk, causes market rejection of grain or reduction in the market price. By contrast, the lower mycotoxin content in GE maize grain can help to minimize the exposure of humans to health hazardous toxins through the diet. The risk of exposure to mycotoxins is particularly severe in developing countries. Under dry and warm conditions maize is grown under drought stress and technological resources and infrastructures for routine food monitoring are lacking; both factors favour the development of toxinogenic fungi^31^. In a climate change scenario with rainfall reduction and increase of temperature, maize will be increasingly subjected to drought stress^32^ and more susceptible to fungal attack^33,34^.

The authorization procedure prior to GE crop cultivation requires the substantial equivalence of composition with non-GE crops as an end point^35^. Apart from mycotoxin levels, our results indicated that the composition of GE maize grain did not differ from that of the isolines for protein, lipid, ADF, NDF and TDF content, and confirm what was found on compositional equivalence between GE crops and non-GE comparators over the last two decades^36^.

### Impact on TOs and NTOs

The European corn borer *Ostrinia nubilalis* (Hubner) (Lepidoptera: Crambidae) and the Mediterranean corn stalk borer *Sesamia nonagrioides* Lefebvre (Lepidoptera: Noctuidae), along with the western corn rootworm (*Diabrotica virgifera virgifera* Le Conte) (Coleoptera: Chrysomelidae) are common pests affecting maize^34^. In our study, only the data on *Diabrotica* spp. abundance were sufficient to perform a reliable meta-analysis. Our results clearly indicated that GE maize was highly effective against *Diabrotica* spp. infestation with 89.7% of pest decrease compared to the non-GE isolines. All data utilised were collected in field experiments where no insecticide was applied. The effectiveness of IR crops against insect pests is the main objective of crop genetic engineering and our data confirm that this target has been achieved, although the use of *Diabrotica* adult number could be regarded as a not entirely reliable indicator, since the damage is mostly caused by larvae. Moreover, the resistance to *Diabrotica* in the last generation maize hybrids is indicated by GE seed producers as a partial one and attempts are ongoing to further improve the resistance trait by using the RNA interference (RNAi) as a novel strategy^13^.

Despite the high effectiveness of IR crops, the evolution of resistance in pests and a consequent reduction of the GE crop effectiveness can not be excluded. Actually, resistance and cross-resistance to Bt maize were recently detected in *Spodoptera frugiperda* (J.E. Smith) (Lepidoptera: Noctuidae) in Puerto Rico^37^, *Busseola fusca* (Fuller) (Lepidoptera: Noctuidae) in South Africa^38^ and in the Coleoptera *D*. *virgifera* in Iowa^39^ even though the implementation of refuges has been mandated in USA, EU, Australia and elsewhere^40^. The refuge strategy, implemented with distinct management practices^41,42^, is based on the idea that refuges, which consist of non-Bt host plants near or in fields of Bt crops, produce susceptible pests that mate with the rare resistant individuals surviving on Bt crops. Another recent approach for delaying the evolution of pest resistance consists in the development of Bt crops expressing more than one Cry toxin, such as the multiple stacked/pyramided Bt crops^43^.

Our study showed that GE maize did not significantly affect the majority of the NTOs families, notably Anthocoridae, Aphididae, Araneae, Carabidae, Chrysopidae, Coccinellidae, Nabidae, Nitidulidae and Staphylinidae. On the contrary, we detected a considerable decrease in Braconidae^44^.

Overall, the results of NTOs are consistent with previous results^17^ showing no effects of IR GE maize on different NT insect taxa, except for the presence of Hymenoptera that was lower in GE maize. Similarly, no effect of Bt maize on 26 arthropod taxa, including herbivores, predators, omnivores, parasitoids and composers, was detected in a meta-analysis of the results from 13 field trials in Spain^19^.

The observed decrease of Braconidae, mostly represented by *M*. *cingulum* (98% of observations), in GE crops is in line with other findings^18^ that showed a decrease of populations belonging to the functional guild of parasitoids. Since the abundance of parasitoids depends largely on the abundance of the target pest host, the observed decrease of *M*. *cingulum* in GE maize is very likely an indirect effect of the decrease in *O*. *nubilalis* caused by the GE maize.

Differently from other results^19^, covering a limited area the NE Iberian Peninsula, we found, on the basis of observations obtained in three continents, an increase in Cicadellidae, although not supported by the sensitivity analysis revealing that this result can not be considered robust.

From a methodological point of view, all the above-cited meta-analyses have taken into consideration a larger number of observations, including experiments not having the appropriate comparators and statistics and embracing the grey literature.

### Impact on biomass decomposition

Plant nutrition and soil quality are directly affected by the decomposition of organic matter, which in turn depends on plant tissue composition, environmental conditions, and soil biota. Our analyses indicated that lignin concentration in leaves and stalks did not change between GE maize and their isolines. Quantity and quality of lignin are considered the main traits affecting the rate of plant biomass decomposition because lignin is the most recalcitrant component of plant tissues and offers protection to associated polysaccharides, proteins, and other plant components more susceptible to biodegradation (*e.g*.,^45–47^). The rates of litter mass loss correlate with the initial lignin and N contents^48,49^. Consistently, we observed no difference in stalk mass loss between GE crops and their isolines. By contrast, we found significant differences in the loss of total biomass that includes all crop residues (leaves, stalks and tassels). This disagreement might be due to differences between GE and their isolines in the proportion and composition of the plant organs in the residue, i.e. stalks and leaves which have a distinct rate of degradation^50,51^. Unfortunately, it was not possible to compare the results of biomass loss with those of CO_2_ soil fluxes and C storage in soil due to an insufficient number of data to be analysed.

Laboratory and greenhouse studies on IR and HT maize have drawn attention to GE proteins in soil and their potential effects on soil biota (*e.g*.,^52,53^), but few studies have evaluated the effects of GE maize on soil biota in field conditions and we could not perform a meta-analysis due to scarcity of data for single taxa or because the data did not fulfil the criteria of meta-analysis. Specifically, field comparisons of GE and non-GE maize revealed sporadic decreases in the biomass of amoebae, earthworms, flagellates, ciliates, as well as of nematodes with no difference or small difference in nematode community composition^54–56^. Therefore, in the case of nematodes that utilise as a food resource bacteria, fungi or plants, GE maize seems to have a direct effect on specific food resources rather than to have an indirect effect^54^. In addition soil microbial biomass and activity did not change between GE and non-GE maize^54,55,57^. Bacterial community profiles in the rhizosphere were not modified or only slightly modified by HT-maize hybrids^55,58^ and IR-maize hybrids^54,55,57^. However, if some slight bacterial community changes occurred, these were shown not to be persistent^55^, probably due to the rapid degradation or inactivation of toxins in soil in field conditions^53^. Finally, the arbuscular mycorrhizal fungal (AMF) community, spore abundance and root colonization did not change in Bt *versus* non-Bt maize, suggesting that the cultivation of Bt maize may not have an impact on AMF in soil under field conditions^59^.

In conclusion, our meta-analysis of 21 years of field data on the agro-environmental impact of GE maize clearly shows the benefits in terms of increases in grain yield and quality, and in decreases of the target insect *Diabrotica* spp. Our analysis highlights modest or no effect on the abundance of non-target insects, suggesting no substantial effect on insect community diversity. This confirms previous results on NTOs and extends our knowledge to new taxa. We provide also strong evidence that GE maize cultivation reduces mycotoxin content in grain. Since mycotoxin contamination in maize grain annually leads to high economic losses in all regions of the world, the protection of maize plants through the use of GE technology against the damage of insects, favouring the development of toxinogenic fungi, can be seen as an effective tool to reduce the contamination of grain. This can lead to increases in economic income and quality of the production and to reductions in the human exposure to mycotoxins, thus reducing health risks. Finally, as GE technology moves forward involving new crops, new traits and new adopting countries, new experimental field data should be made available in an open and standardized format allowing researchers and regulators to draw further conclusions on the agro-environmental and health risks of GE crops.

## Methods

### Literature survey and database construction

A database of the effects of GE maize on crop yield and quality, TOs and NTOs (plants, animals, microbes), and biogeochemical cycles was built by surveying peer-reviewed literature within the Web of Science^TM^ Core Collection database (Clarivate Analytics). The search, covering the period from November 1996 to September 2016, aimed at identifying articles that reported agro-environmental effects of GE maize *vs*. non-GE comparator (near isogenic line) grown in field conditions. The search phrases used were (maize OR corn OR *Zea mays*) AND (genetically modified OR GMO OR transgenic OR GM OR Bt OR genetically engineered OR comparator OR near-isogenic OR herbicide tolerant OR insect resistant). Selected Web of Science^TM^ categories were: plant sciences, biotechnology applied microbiology, agronomy, entomology, agriculture multidisciplinary, environmental sciences, horticulture, ecology, soil science, microbiology, zoology, environmental studies and biodiversity conservation. Document types were articles and reviews. Languages were: English, Spanish, French, and German. Experiments performed in controlled environments (greenhouse or climatic chamber) were excluded. We selected experiments performed in field conditions whose controls were represented by near isogenic lines and that were managed in the same way as the corresponding GE maize. We accepted only pairwise comparisons in which controls entailed non-GE varieties grown under identical conditions, because our aim was to elucidate the effect of genetic engineering of maize on agro-environmental traits. When studies include measures of both peak abundance (highest density on any given sample dates) and seasonal abundance (averaged over multiple sample dates) the peak data should be retained. To be included in the database, articles needed to report data on the agro-environmental impact of GE maize with a measure of the variance (standard deviation, standard error, coefficient of variation, least significant difference) or the statistical significance of the analysis (t- or *P*-value), and the sample size. Data were classified into five categories: (1) grain yield and quality including economic parameters; (2) NTOs, (3) TOs; (4) biogeochemical cycles; (5) biodiversity. For all categories, from each article data were extracted as shown in Supplementary 1 Table 9. Data from figures were extracted by the GraphGrabber software package^60^.

The following information was also extracted: (i) geographic location (latitude and longitude); (ii) transformation event(s), expressed toxin(s), hybrid name, commercial name, seed company in GE hybrid, (iii) hybrid name, commercial name, seed company in non-GE isoline; (iv) treatment (*e.g*., location, year of cultivation, irrigation, biocontrol); (v) NTO taxon. For each NTO taxon, phylum, class, order, and family were determined and included in the database. Non-target organisms were classified according to the Fauna Europaea (http://www.fauna-eu.org) and the Integrated Taxonomic Information System (http://www.itis.gov). In addition, for the categories grain yield and quality and NTOs we implemented our datasets following the comparison with available datasets published by Klümper and Qaim^16^ and Wolfenbarger *et al*.^18^, respectively. Klümper and Qaim^16^ reported aggregated data for soybean, maize, and cotton and we disaggregated these data to obtain the ones referred only to maize. Wolfenbarger *et al*.^18^ included data requested from Authors in their dataset, and this allowed us to include 40 observations in this study.

For articles that reported observations from more than one treatment, all the observations were included in the database. To perform reliable analyses, agro-environmental traits were included in the analysis if they were reported in at least three articles. For each trait included in the four categories, a dataset was built to perform the calculation of the number of studies and observations and the statistical analysis described as follows. Whenever possible, we analyzed the trait for the classes single, double and triple stacks, and expressed toxin.

The methods of analysis accepted for grain quality, residues mass loss and CO_2_ emission from residues and soil are given in Supplementary 1 Table 8.

### Response ratios and statistical analysis

Effect size was estimated by Hedges’ *g*^61^. For each observation included in the database, Hedges’ *g* was calculated for all traits as the difference between the mean of GE and non GE maize divided by the pooled standard deviation and adjusted by a weighting factor based on the number of replicates per treatment. The Hedges’ *g* was calculated using the Comprehensive Meta-Analysis software, version 3^62,63^, starting from means, SD or *P* values and number of replicates. Hedge’ *g* is a unitless index that ranges from −∞ to +∞ and estimates the size and direction of impact. Hedges’ *g* values of zero signify no difference in the measured trait due to genetic engineering, while positive and negative values imply an increase and a decrease in the trait following genetic engineering, respectively. For each trait we calculated the mean effect size (*g*^+^) across the observations from all articles, weighted by sample size. Data were analyzed using a random-effect model, assuming that the true effect sizes could vary from study to study^62^. Under this model, two sources of variance are taken into account and within-study error and across-study error are calculated. The variability within- and across-studies is assumed to have a normal distribution. The model is hierarchical because the within-study variance is nested within the among-study variability and is mixed because it has more than one variance. To test whether g^+^ differed significantly from zero (i.e., no change due to genetic engineering), we assessed whether the 95% bootstrap-confidence interval (CI) of bias-corrected g^+^ did not overlap zero based on 999 iterations^62^. We also tested whether effect sizes across the observations from all articles were homogeneous, using the Q total statistic (Q_t_) based on a chi-squared test^62^. A significant Q_t_ indicates that the variance among effect sizes is greater than that expected from sampling error alone. The calculations were made using the Comprehensive Meta-Analysis software, version 3. The mean percentage of change for the g^+^ values different from zero was calculated as [exp(R^+^) −1] × 100 where R^+^ is the weighted mean response ratio (R) across the observations from all articles^62^. The response ratio was calculated as ln (X_GE_/X_non-GE_) where X_GE_ is the trait in the GE maize and X_non-GE_ is the trait in the isoline.

To verify whether the results would change if a set of observations was removed from the analysis, we applied the sensitivity analysis described by Borenstein *et al*.^63^. This analysis was performed for each trait using a reduced dataset obtained by randomly removing one observation per article, when the number of observations per article was higher than one. Thus, for each trait, the mean effect size obtained from the reduced dataset was compared with the one obtained from whole dataset. This procedure is available in the software Comprehensive Meta-Analysis software, version 3. Comparisons were made using 95% bootstrap CIs based on 999 iterations and using *P*-values. The data analyses were performed using the Comprehensive Meta-Analysis software, version 3.

In order to identify the publication bias due to the tendency for journals to only publish studies with statistically significant results, sensitivity analysis was performed comparing the fail-safe number with a threshold calculated as 5n + 10, where n is the original number of studies^64^. A fail-safe number is considered robust if it is greater than the threshold value. The calculation of fail-safe number was done applying a random-effect model in the Comprehensive Meta-Analysis version 3.

### Data availability

All data generated or analyzed during this study are included in this published article (and its Supplementary files).

## Electronic supplementary material

Dataset 1

Dataset 2

Dataset 3

Dataset 4

Dataset 5

## References

[1] Snow AA, Palma PM (1997). Commercialization of transgenic plants: potential ecological risks. Bioscience.

[2] Benbrook CM (2012). Impacts of genetically engineered crops on pesticide use in the U.S. - the first sixteen years. Environ. Sci. Eur..

[3] FAO *FAO Statistical Pocketbook 2015* (FAO, 2015).

[4] ISAAA *Global Status of Commercialized Biotech/GM Crops: 2016*. *ISAAA Brief No*. *52* (ISAAA, 2016).

[5] Aldemita RR, Reaño IME, Solis RO, Hautea RA (2015). Trends in global approvals of biotech crops (1992–2014). GM crops & food.

[6] Ortego, F., Pons, X., Albajes, R. & Castañera, P. European Commercial Genetically Modified Plantings and Field Trials In *Environmental Impact of Genetically Modified Crops* (eds Ferry, N. & Gatehouse, A. M. R.) 327–343 (CAB International, 2009).

[7] Qaim M (2009). The economics of genetically modified crops. Ann. Rev. Res. Econ..

[8] Burachik M (2010). Experience from use of GMOs in Argentinian agriculture, economy and environment. New Biotechnol..

[9] Arthur GD (2011). Benefits and concerns surrounding the cultivation of genetically modified crops in Africa: The debate. African J. Biotechnol..

[10] Xu Z, Hennessy DA, Sardana K, Moschini GC (2013). The realized yield effect of genetically engineered crops: U.S. maize and soybean. Crop Sci..

[11] Nicolia A, Manzo A, Veronesi F, Rosellini D (2014). An overview of the last 10 years of genetically engineered crop safety research. Crit. Rev. Biotechnol..

[12] Wang F, Peng S, Cui K, Nie L, Huang J (2014). Field performance of Bt transgenic crops: A review. Austr. J. Crop Sci..

[13] U.S. National Academies of Sciences, Engineering, and Medicine *Genetically Engineered Crops: Experiences and Prospects*. (The National Academies Press, 2016). 28230933

[14] Finger R (2011). A meta-analysis on farm-level costs and benefits of GM crops. Sustainability.

[15] Areal FJ, Riesgo L, Rodríguez-Cerezo E (2013). Economic and agronomic impact of commercialized GM crops: a meta-analysis. J. Agric. Sci..

[16] Klümper W, Qaim M (2014). A meta-analysis of the impacts of genetically modified crops. PLoS One.

[17] Marvier M, McCreedy C, Regetz J, Kareiva P (2007). A meta-analysis of effects of Bt cotton and maize on nontarget invertebrates. Science.

[18] Wolfenbarger LL, Naranjo SE, Lundgren JG, Bitzer RJ, Watrud LS (2008). Bt crop effects on functional guilds of non-target arthropods: a meta-analysis. PLoS One.

[19] Comas, C., Lumbierres, B., Pons, X. & Albajes R. No effects of *Bacillus thuringiensis* maize on non-target organisms in the field in southern Europe: a meta-analysis of 26 arthropod taxa. *Transgenic Res*. **23** (2014). 10.1007/s11248-013-9737-0 23904218

[20] Naranjo, S. E. Impacts of Bt crops on non-target invertebrates and insecticide use patterns *CAB Reviews* **11** (2009).

[21] Ercoli L, Masoni A, Pampana S, Arduini I (2007). Allelopathic effects of rye, brown mustard and hairy vetch on redroot pigweed, common lambsquarter and knotweed. Allelopathy J..

[22] Ercoli L, Lulli L, Arduini I, Mariotti M, Masoni A (2011). Durum wheat grain yield and quality as affected by S rate under Mediterranean conditions. Eur. J. Agron..

[23] Lescourret F (2015). A social–ecological approach to managing multiple agro-ecosystem services. Curr. Opin. Env. Sust..

[24] Fernandez-Cornejo, J., Wechsler, S., Livingston, M., & Mitchell, L. Genetically engineered crops in the United States. ERR-162 U.S. Department of Agriculture, Economic Research Service, February 2014.

[25] Oerke EC (2006). Crop losses to pests. J. Agr. Sci..

[26] Gianessi LP (2013). The increasing importance of herbicides in worldwide crop production. Pest Manag. Sci..

[27] Brookes G, Barfoot P (2013). Key environmental impacts of global genetically modified (GM) crop use 1996–2011. GM crops & food.

[28] Wu F (2006). Mycotoxin reduction in *Bt* corn: potential economic, health, and regulatory impacts. Transgenic Res..

[29] Ostry V (2010). A review on comparative data concerning Fusarium mycotoxins in Bt maize and non-Bt isogenic maize. Mycotox. Res..

[30] Abbas HK (2013). Implications of Bt traits on mycotoxin contamination in maize: overview and recent experimental results in Southern United States. J. Agr. Food Chem..

[31] Strosnider H (2006). Workgroup report: public health strategies for reducing aflatoxin exposure in developing countries. Environ. Health Persp..

[32] Vallebona C, Pellegrino E, Frumento P, Bonari E (2015). Temporal trends in extreme rainfall intensity and erosivity in the Mediterranean region: a case study in southern Tuscany, Italy. Climatic Change.

[33] Rosenzweig C, Iglesias A, Yang XB, Epstein PR, Chivian E (2001). Climate change and extreme weather events; implications for food production, plant diseases, and pests. Glob. Change Hum. Health.

[34] Meinke LJ (2009). Western corn rootworm (*Diabrotica virgifera virgifera* LeConte) population dynamics. Agric. Forest Entomol.

[35] EFSA Panel on Genetically Modified Organisms (GMO). Guidance on the environmental risk assessment of genetically modified plants. *EFSA J*. **8**, 1879–1990 (2010).

[36] Herman RA, Price WD (2013). Unintended compositional changes in genetically modified (GM) crops: 20 years of research. J. Agric. Food Chem..

[37] Storer NP (2010). Discovery and characterization of field resistance to Bt maize: *Spodoptera frugiperda* (Lepidoptera: *Noctuidae*) in Puerto Rico. J. Econ. Entomol..

[38] van Rensburg JBJ (2007). First report of field resistance by the stem borer, *Busseola fusca* (Fuller) to Bt-transgenic maize. S. Afr. J. Plant Soil.

[39] Gassmann AJ, Petzold-Maxwell JL, Keweshan RS, Dunbar MW (2011). Field-evolved resistance to Bt maize by western corn rootworm. PloS One.

[40] Tabashnik BE (2008). Delaying insect resistance to transgenic crops. Proc. Natl. Acad. Sci..

[41] Gould F (1998). Sustainability of transgenic insecticidal cultivars: integrating pest genetics and ecology. Annu. Rev. Entomol..

[42] Tabashnik BE, Brévault T, Carrière Y (2013). Insect resistance to Bt crops: lessons from the first billion acres. Nat. Biotechnol..

[43] Carrière Y, Crickmore N, Tabashnik BE (2015). Optimizing pyramided transgenic Bt crops for sustainable pest management. Nat. Biotechnol..

[44] Watanabe C (1967). Further revision of the genus *Macrocentrus Curtis* in Japan, with descriptions of two new species (Hymenoptera, Braconidae). Insecta Matsumurana.

[45] Tovar-Gomez MR, Emile JC, Michalet-Doreau B, Barriere Y (1997). *In situ* degradation kinetics of maize hybrid stalks. Anim. Feed Sci. Tech..

[46] Hopkins DW, Gregorich EG (2003). Detection and decay of the Bt endotoxin in soil from a field trial with genetically modified maize. Eur. J. Soil Sci..

[47] Flores S, Saxena D, Stotzky G (2005). Transgenic Bt plants decompose less in soil than non-Bt plants. Soil Biol. Biochem..

[48] Aber JD, Melillo JM (1982). Nitrogen immobilization in decaying hardwood leaf litter as a function of initial nitrogen and lignin content. Can. J. Botany.

[49] Melillo JM, Aber JD, Muratore FF (1982). Nitrogen and lignin control of hardwood leaf litter decomposition dynamics. Ecology.

[50] Tarkalson DD, Kachman SD, Knops JM, Thies JE, Wortmann CS (2008). Decomposition of Bt and non-Bt corn hybrid residues in the field. Nutr. Cycl. Agroecosys..

[51] Yanni SF, Whalen JK, Ma BL (2010). Crop residue chemistry, decomposition rates, and CO_2_ evolution in Bt and non-Bt corn agroecosystems in North America: a review. Nutr. Cycl. Agroecosys..

[52] Saxena D, Stotzky G (2001). *Bacillus thuringiensis* (Bt) toxin released from root exudates and biomass of Bt corn has no apparent effect on earthworms, nematodes, protozoa, bacteria, and fungi in soil. Soil Biol. Biochem..

[53] Icoz I, Stotzky G (2008). Fate and effects of insect-resistant Bt crops in soil ecosystems. Soil Biol. Biochem..

[54] Griffiths BS (2005). A comparison of soil microbial community structure, protozoa and nematodes in field plots of conventional and genetically modified maize expressing the *Bacillus thuringiensis* CryIAb toxin. Plant Soil.

[55] Griffiths BS (2007). Microbial and microfaunal community structure in cropping systems with genetically modified plants. Pedobiologia.

[56] Krogh PH (2007). Responses by earthworms to reduced tillage in herbicide tolerant maize and Bt maize cropping systems. Pedobiologia.

[57] Devare MH, Jones CM, Thies JE (2004). Effect of Cry3Bb transgenic corn and tefluthrin on the soil microbial community. J. Environ. Qual..

[58] Schmalenberger A, Tebbe CC (2002). Bacterial community composition in the rhizosphere of a transgenic, herbicide-resistant maize (*Zea mays*) and comparison to its non-transgenic cultivar Bosphore. FEMS Microbiol. Ecol..

[59] Cheeke TE, Cruzan MB, Rosenstiel TN (2013). Field evaluation of arbuscular mycorrhizal fungal colonization in *Bacillus thuringiensis* toxin-expressing (Bt) and non-Bt maize. Appl. Environ. Microb..

[60] Quintessa Graph Grabber. Quintessa Limited, Oxfordshire, UK. (2009).

[61] Hedges L (1981). Distribution theory forGlass’s estimator of effect size and related estimators. J. Educ. Stat..

[62] Borenstein, M., Hedges, L. V., Higgins, J. P. T. & Rothstein, H. R. Comprehensive meta-analysis version 3. Englewood, NJ: Biostat. (2014).

[63] Borenstein, M., Hedges, L. V., Higgins, J. P. T. & Rothstein, H. R. *Introduction to Meta-Analysis* (Wiley, 2009).

[64] Rosenberg MS (2005). The file-drawer problem revisited: a general weighted method for calculating fail-safe numbers in meta-analysis. Evolution.

