# Author Correction: Impact of genetically engineered maize on agronomic, environmental and toxicological traits: a meta-analysis of 21 years of field data

**DOI:** 10.1038/s41598-018-24840-y

**Published:** 2018-04-19

**Authors:** Elisa Pellegrino, Stefano Bedini, Marco Nuti, Laura Ercoli

**Affiliations:** 1 Institute of Life Sciences, Scuola Superiore Sant’Anna, Piazza Martiri della Libertà 33, 56127 Pisa, Italy; 2 Department of Agriculture, Food and Environment, University of Pisa, via del Borghetto 80, 56125 Pisa, Italy

Correction to: *Scientific Reports* 10.1038/s41598-018-21284-2, published online 15 February 2018

This Article contains an error in the legend of Figure 3.

“Response ratios. Effects of GE maize hybrids on the significant traits: grain yield and damaged ears (a), grain quality (fumonisins, thricotecenes, mycotoxins), target (*Diabrotica* spp.) and non-target (*Braconidae*, *Cicadellidae*) organisms and residue mass loss (b). The response ratio was calculated as the mean percentage of change for the weighted Hedge’s *g* (g+) values different from zero between the GE hybrids and their isolines. SS = single eventhybrid; DS = double stacked hybrid; TS = triple stacked hybrid; QS = quadruple stacked hybrid.”

should read:

“Response ratios. Effects of GE maize hybrids on the significant traits: grain yield and damaged ears (a), grain quality (fumonisins, trichothecene, mycotoxins), target (*Diabrotica* spp.) and non-target (*Braconidae*, *Cicadellidae*) organisms and residue mass loss (b). The response ratio was calculated as the mean percentage of change for the weighted Hedge’s *g* (g+) values different from zero between the GE hybrids and their isolines. SS = single eventhybrid; DS = double stacked hybrid; TS = triple stacked hybrid; QS = quadruple stacked hybrid.”

